# vPE: Toward a new generation of error-free prime editing

**DOI:** 10.1016/j.omtn.2026.102898

**Published:** 2026-03-23

**Authors:** Mahnoor Jamil, Ambreen Zahra, Rizwana Maqbool, Muhammad Imran Arshad, Sultan Habibullah Khan

**Affiliations:** 1 Center for Advanced Studies in Agriculture and Food Security, University of Agriculture, Faisalabad, Pakistan; 2 Center of Agricultural Biochemistry and Biotechnology, University of Agriculture, Faisalabad, Pakistan; 3 Department of Plant Breeding and Genetics, University of Agriculture, Faisalabad, Pakistan; 4 Department of Veterinary Preventive Medicine, College of Veterinary Medicine, Qassim University, Buraydah 52571, Kingdom of Saudi Arabia

## Abstract

Prime editing is a precise “search and replace” genome editing tool that enables programmed genome modifications without the need for double-stranded breaks or additional repair templates. However, the retention of the unedited 5′ strand hinders the incorporation of stable edits into the genome, and the rate of indel errors is also high. Recently, *Chauhan* et al. reported an engineered prime editor (PE), i.e., a very precise PE (vPE), that introduces nick-relaxing mutations, which can favor stable genome edits by destabilizing the 5′ end and degrading the nicked end, thereby minimizing indel errors. This vPE holds promising potential as a transformative force in advancing human disease therapeutics and enabling innovative breakthroughs in crop improvement.

## Current prime editing toolkit

The ever-expanding toolbox of genome editing enables researchers to manipulate genetic information with greater specificity and efficiency. Over the past decade, scientists have engineered a remarkable array of genome editing tools, including nucleases, base editors, and prime editors (PEs), introducing new variants with higher precision and continually improving editing efficiencies. PEs are CRISPR-based tools capable of introducing highly specific changes in the genome by creating a nick, in contrast to the unpredictable mutations that arise following repair of double-strand breaks in conventional CRISPR-Cas9 editing. PEs offer an invaluable genome editing platform characterized by high specificity and precision, enabling a wide array of edits, including single or multiple base substitutions, small insertions, and small deletions, thereby providing strong control over the modifications required at the target site. Scientists have made substantial efforts by optimizing the architecture of pegRNAs, protein domains, and inhibiting cellular mismatch repair machinery to develop a series of PEs, including PE1, PE2, PE4, ePPE, twinPE, and many more, with each new version circumventing the limitations of its predecessor.^1^ However, some limitations of prime editing remain. These include competition between the unedited 5′ DNA flap and the 3′ DNA flap containing the desired edits, as well as the generation of indels in a fraction of targeted cells at non-target sites, which can have deleterious effects.^2^ Thus, there is a need for a more precise and highly efficient prime editing system that can address these challenges.

## vPE: A new player in prime editing

Recently, *Chauhan* et al. have reported a series of PEs by engineering the Cas9 nuclease to address these challenges, paving the future of highly targeted, specific, and low error-prone genome editing.^3^ In this study, a new mechanism for increasing PE fidelity was reported, establishing a strong correlation between relaxed nick positioning, degradation of the unedited 5′ strand, reduced indel errors, and improved editing efficiency. To address the issue of retention of the unedited 5′ DNA strand, the authors screened different alanine substitutions in the DNA-binding domain of *Streptococcus pyogenes* Cas9. They successfully found that some of these Cas9 variants exhibited relaxed nick positioning. Among these mutations, R976A and H982A showed significant 5′ end destabilization. Furthermore, several mutations (R780A, K810A, K848A, K855A, R976A, and H982A) also showed improvement in edit-to-indel ratios by up to 10-fold and reduced indel errors by 20-fold through nick positioning relaxation and 5′ end degradation. The combination of these mutations in PE drastically improved editing efficiencies and lowered indel errors. These results led to the development of a precise PE (pPE) with two engineered mutations, K848A-H982A, which reduced indel errors by as much as 36-fold and improved the edit-to-indel ratio by up to 28-fold. Comparison of different modes of prime editing, i.e., pegRNA only and pegRNA+ngRNA in pPE with PEmax, also showed significant improvements in the efficacy of pPE through reduced indels. However, suppression of indel errors by pPE displays a modest trade-off in editing efficiency compared with PEmax. The pPE was further improved by incorporating mutations that boost Cas9 activity. After assessing several individual mutations and their combinations, one variant, R221K-K848A-H982A-N1317R, showed the greatest improvement in edit-to-indel ratios, increasing from 276:1 to 354:1. This improved version of PE, with higher editing efficiency and a reduced indel error rate, was named extra-pPE (xPE). Although xPE showed a significant improvement in editing efficiency compared with pPE, it was still lower than PEmax.^3^ Since the mutations in xPE are not known to reduce Cas9 activity,^4^ efforts were directed toward identifying the impact of nick repositioning on Cas9 function. The Cas9 in xPE was swapped with the Cas9 in PE7, an efficiency-boosting PE reported to stabilize pegRNA through incorporation of the La poly-U RNA binding protein,^5^ resulting in the development of a very precise PE (vPE). This vPE, engineered by combining features of different PEs, is characterized by lower indel errors and improved editing efficiencies compared with PEmax.^3^ Furthermore, analysis of this vPE in different mammalian cell lines revealed similar efficiencies, confirming its precision and high efficacy while minimizing the indel errors associated with previous PEs in correcting various edits.

## Scope of vPE applications in human therapeutics and agriculture

**Figure 1:**
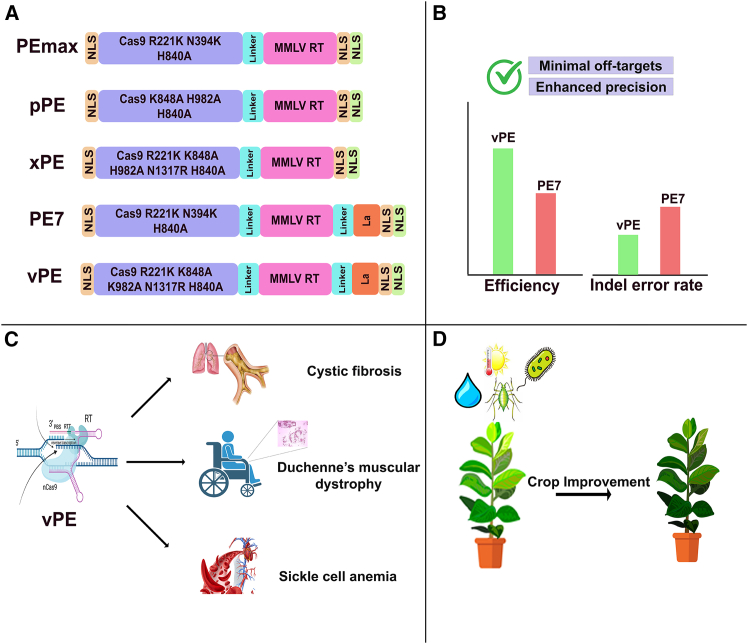
Very-precise prime editors (vpPEs) in therapeutics and crop biotechnology (A) Structure of different types of prime editors. (B) Performance characteristics of vpPEs. (C) Therapeutic potential of vpPEs. (D) Applications in agriculture.

This discovery of vPE holds promising potential in human therapeutics and crop improvement (Figure 1). CRISPR systems, particularly PEs, have already been reported to cure some genetic disorders, including sickle cell disease, Tay-Sachs disease, cystic fibrosis, and many others, but low editing efficiencies have been a serious concern for therapeutic purposes.^6^^,^^7^^,^^8^ Therefore, the vPE developed by *Chauhan* et al. holds significant potential for increasing editing efficiencies and curing these diseases with higher precision and reduced off-target effects. Moreover, it can contribute to the correction of some genetic disorders, e.g., Duchenne muscular dystrophy (DMD), spinal muscular atrophy, and Hemophilia A, which need efficient insertion or deletion of large DNA fragments without special requirements, unlike previous editing systems, including PASTE and PE-PASSIGE, which rely on additional enzymatic components to treat these diseases.^9^^,^^10^^,^^11^ Although this system has so far been demonstrated only in human cell lines, its design principles and advantages can be directly translated to plant genome editing applications, offering higher edit-to-indel ratios. Previous versions of PEs showed very low editing efficiency in polyploid crops.^12^ So, this newly introduced vPE can be utilized to achieve genome editing with higher efficiency in polyploid crops for trait improvement. Moreover, it can be optimized for multiplex genome editing using transfer RNA-processed polycistronic RNA.^13^ One promising potential of this PE is highly precise and targeted genome editing in plants via delivery of prime editing reagents through viral vectors and nanoparticles, avoiding labor-intensive, tissue culture-dependent methods. Furthermore, the existing architecture of vPE can be extended to other Cas variants to improve their editing efficiencies. vPE can be further modified for organellar-specific prime editing by incorporating design principles of CyDENT, a base editor targeting mitochondrial and chloroplast genomes.^14^ Conclusively, this PE expands the existing genome editing toolbox and offers new avenues for human therapeutics and crop improvement. However, further investigations are needed to optimize this system and expand its applications in plant and human biology.

## Declaration of interests

The authors do not have any competing interests and did not receive any external funding.

## References

[1] Chen P.J., Liu D.R. (2023). Prime editing for precise and highly versatile genome manipulation. Nat. Rev. Genet..

[2] Fiumara M., Ferrari S., Omer-Javed A., Beretta S., Albano L., Canarutto D., Varesi A., Gaddoni C., Brombin C., Cugnata F. (2024). Genotoxic effects of base and prime editing in human hematopoietic stem cells. Nat. Biotechnol..

[3] Chauhan V.P., Sharp P.A., Langer R. (2025). Engineered prime editors with minimal genomic errors. Nature.

[4] Spencer J.M., Zhang X. (2017). Deep mutational scanning of S. pyogenes Cas9 reveals important functional domains. Sci. Rep..

[5] Yan J., Oyler-Castrillo P., Ravisankar P., Ward C.C., Levesque S., Jing Y., Simpson D., Zhao A., Li H., Yan W. (2024). Improving prime editing with an endogenous small RNA-binding protein. Nature.

[6] Anzalone A.V., Randolph P.B., Davis J.R., Sousa A.A., Koblan L.W., Levy J.M., Chen P.J., Wilson C., Newby G.A., Raguram A., Liu D.R. (2019). Search-and-replace genome editing without double-strand breaks or donor DNA. Nature.

[7] Geurts M.H., de Poel E., Pleguezuelos-Manzano C., Oka R., Carrillo L., Andersson-Rolf A., Boretto M., Brunsveld J.E., van Boxtel R., Beekman J.M., Clevers H. (2021). Evaluating CRISPR-based prime editing for cancer modeling and CFTR repair in organoids. Life Sci. Alliance.

[8] Zahra A., Sajjal S., Bilal H.A., Khan A., Arshad M.I. (2025). Personalized CRISPR-Based K-abe Therapy Using Genetic Scissors. Hum. Gene Ther..

[9] Yarnall M.T.N., Ioannidi E.I., Schmitt-Ulms C., Krajeski R.N., Lim J., Villiger L., Zhou W., Jiang K., Garushyants S.K., Roberts N. (2023). Drag-and-drop genome insertion of large sequences without double-strand DNA cleavage using CRISPR-directed integrases. Nat. Biotechnol..

[10] Pandey S., Gao X.D., Krasnow N.A., McElroy A., Tao Y.A., Duby J.E., Steinbeck B.J., McCreary J., Pierce S.E., Tolar J. (2025). Efficient site-specific integration of large genes in mammalian cells via continuously evolved recombinases and prime editing. Nat. Biomed. Eng..

[11] Shahid A., Zahra A., Aslam S., Shamim A., Ali W.R., Aslam B., Khan S.H., Arshad M.I. (2026). Appraisal of CRISPR technology as an innovative screening to therapeutic toolkit for genetic disorders. Mol. Biotechnol..

[12] Lin Q., Zong Y., Xue C., Wang S., Jin S., Zhu Z., Wang Y., Anzalone A.V., Raguram A., Doman J.L. (2020). Prime genome editing in rice and wheat. Nat. Biotechnol..

[13] Waqas M.A.B., Awan M.J.A., Amin I., Arif M., Mukhtar Z., Mansoor S. (2025). Engineering high yield basmati rice by editing multiple negative regulators of yield. Mol. Biol. Rep..

[14] Jamil M., Zahra A., Maqbool R., Arshad M.I., Khan S.H. (2025). CyDENT: A tale of CRISPR-free strand-specific base editor for nuclear and organellar genomes. Mol. Ther. Nucleic Acids.

